# Retraction: Medical Emergency Resource Allocation Model in Large-Scale Emergencies Based on Artificial Intelligence: Algorithm Development

**DOI:** 10.2196/98669

**Published:** 2026-04-28

**Authors:** JMIR Publications Editorial Office

**Affiliations:** 1 JMIR Publications Toronto, ON

The JMIR Publications Editorial Office is retracting the article:

Medical Emergency Resource Allocation Model in Large-Scale Emergencies Based on Artificial Intelligence: Algorithm Development [1]

This action is being taken due to identified concerns regarding potential manipulation of the submission process and the relevance of several references.

The concerns with this publication were identified during an investigation of a series of articles with related concerns. Author Lin Du was contacted and offered an opportunity to comment on these findings and the proposed retraction but did not respond to any attempts at communication.
